# Fano effect and bound state in continuum in electron transport through an armchair graphene nanoribbon with line defect

**DOI:** 10.1186/1556-276X-8-330

**Published:** 2013-07-22

**Authors:** Wei-Jiang Gong, Xiao-Yan Sui, Yan Wang, Guo-Dong Yu, Xiao-Hui Chen

**Affiliations:** 1 College of Sciences, Northeastern University, Shenyang 110004, China; 2 Department of Physics, Jilin University, Changchun 130023, China

**Keywords:** Fano effect, Bound state in continuum, Graphene nanoswitch

## Abstract

Electron transport properties in an armchair graphene nanoribbon are theoretically investigated by considering the presence of line defect. It is found that the line defect causes the abundant Fano effects and bound state in continuum (BIC) in the electron transport process, which are tightly dependent on the width of the nanoribbon. By plotting the spectra of the density of electron states of the line defect, we see that the line defect induces some localized quantum states around the Dirac point and that the different localizations of these states lead to these two kinds of transport results. Next, the Fano effect and BIC phenomenon are detailedly described via the analysis about the influence of the structure parameters. According to the numerical results, we propose such a structure to be a promising candidate for graphene nanoswitch.

**PACS:**

81.05.Uw, 71.55.-i, 73.23.-b, 73.25.+i

## Introduction

Since 2004, the monolayer graphene has been successfully realized in experiment [1,2]. Subsequently, its intriguing properties originating from the strictly two-dimensional structure and massless Dirac fermion-like behavior of low-energy excitation have attracted intensive attention [3,4]. Graphene can be tailored into various edge nanoribbons. Their semiconducting properties with a tunable band gap dependent on the structural size and geometry make them good candidates for the electric and spintronic devices [5]. Due to this reason, the graphene nanoribbons (GNRs) become of particular interest. According to the edge termination types, the GNRs are generally classified into two basic groups, i.e., the armchair and zigzag GNRs [6-8]. In the tight-binding model with nearest-neighbor approximation, the zigzag GNRs are always metallic and exhibit spin-polarized edge states [6-8]. Instead, the armchair GNRs (AGNRs) show metallic characteristics when only *M*=3*n*+2 (*M* denotes its width with *n*∈*integer*), whereas they are semiconducting otherwise [7-9]. Due to the advance and development of experiment, the GNRs can be successfully manufactured by different approaches, such as the high-resolution lithography and etching technique [10,11], chemical means [12,13], or the unzipping of carbon nanotubes [14,15]. Besides, graphene field-effect transistors have been experimentally realized by making use of the band gap introduced in GNRs [12,16]. These experimental progress encourage theoretical researchers to further pay attention to the electric or magnetic properties of the GNRs or GNR heterojunctions [17-23].

Because of the presence of dislocations, microcracks, grain boundaries, and phase interfaces in their growth, experimentally obtained graphene samples are not always single-crystalline materials. These abnormal mechanisms cause some significant physics properties of graphene [24-28]. Recently, a peculiar topological line defect in graphene was reported experimentally by Lahiri [29]. This topological line defect is created by alternating the Stone-Thrower-Wales defect and divacancies, leading to a pattern of repeating paired pentagons and octagons [30]. It was found that this line defect has metallic characteristics. Following this work, some groups proposed a valley filter based on the scattering of this line defect in graphene [31]. Next, using a tight-binding model calculation, Bahamon et al. have observed the metallic characteristics and Fabry-P’erot oscillation phenomena in graphene line defects [32]. After these works, researchers dedicated themselves to the discussion about the electronic and magnetic properties of graphene with a topological line defect; the line defect-based electronics has been gradually established [33-36]. Then, the influence of the line defect on the electron properties of the GNRs have become one main concern of such a field. Song et al. studied a line defect in zigzag GNR where a bulk energy gap is opened by sublattice symmetry breaking [37]. They found that a gapless state is for a configuration which holds a mirror symmetry with respect to the line defect. Lin and Ni reported that the edge-passivated zigzag GNRs with the line defects along the edge show half-metallicity as the line defect is close to one edge [38]. On the other hand, it has been reported that the topological line defects in the zigzag GNR can induce the tuning of antiferromagnetism to ferromagnetism. Hu et al. found that the applied strain induces the local magnetic moments on the line defect, whose coupling with those on the edges leads to a turnover of the spin polarization on one edge, making the zigzag GNR become a ferromagnetic metal at a large enough strain [39]. In addition, Lü et al. calculated the band structure of a zigzag GNR with line defect [40]. They observed that the lowest conduction subband of this structure connects two inequivalent Dirac points with flat dispersion, which is reminiscent of the flat-bottomed subband of a zigzag GNR. Accordingly, a valley filtering device based on a finite length line defect in graphene was proposed.

It is easy to note that the effect of the line defect in the zigzag GNRs has extensively discussed, but few works focused on the AGNRs with line defect. The main reason may be that the line defect can be extended along the zigzag GNRs. It should be certain that the line defect in the AGNRs plays a nontrivial role in the electron transport manipulation despite its terminated topology. With this idea, we, in this work, investigate the electron transport in an AGNR with line defect. We observe that the line defect induces the abundant Fano effects and BIC phenomenon in the electron transport process, which is tightly dependent on the width of the AGNR. According to the numerical results, we propose such a structure to be a promising candidate for electron manipulation in graphene-based material.

## Model and Hamiltonian

We describe the structure of the AGNR with an embedded line defect using the tight-binding model with the nearest-neighbor approximation, i.e.:

(1) $$H=H_{C}+H_{D}+H_{T},$$

where *H*_*C*_ and *H*_*D*_ are the Hamiltonians of the AGNR and the line defect, respectively. *H*_*T*_ represents the coupling between the AGNR and the defect. These three terms are written as follows:

$$\begin{array}{lll} H_{C} & =\underset{i_{c}}{\sum }\varepsilon_{c}c_{i_{c}}^{†}c_{i_{c}}-t_{0}\underset{\langle i_{c},j_{c}\rangle }{\sum }c_{i_{c}}^{†}c_{j_{c}},\; & \;\\ H_{D} & =\underset{m_{d}}{\sum }\varepsilon_{d}c_{m_{d}}^{†}c_{m_{d}}-t_{D}\underset{\langle m_{d},n_{d}\rangle }{\sum }c_{m_{d}}^{†}c_{n_{d}},\; & \;\\ H_{T} & =-t_{T}\underset{\langle i_{c},m_{d}\rangle }{\sum }(c_{i_{c}}^{†}c_{m_{d}}+\mathrm{H.c.}).\; & \; \end{array}$$

Here, the index *i*_*c*_ (*m*_*d*_) is the site coordinate in the AGNR (line defect), and 〈*i*_*c*_,*j*_*c*_〉 (〈*m*_*d*_,*n*_*d*_〉) denotes the pair of nearest neighbors. *t*_0_ and *t*_*D*_ are the hopping energies of the AGNR and line defect, respectively. *ε*_*c*_ and *ε*_*d*_ are the on-site energies in the AGNR and the line defect, respectively. *t*_*T*_ denotes the coupling between the AGNR and line defect.

With the help of the Landauer-Büttiker formula [41], the linear transport properties in this structure can be evaluated, i.e.:

(2) $$G=\frac{2e^{2}}{h}T(\varepsilon_{F}).$$

*T*(*ω*) is the transmission probability, and *ε*_*F*_ is the Fermi energy. The transmission probability is usually calculated by means of the nonequilibrium Green function technique or the transfer matrix method. In this work, we would like to use the nonequilibrium Green function technique to investigate the electron transport properties. For convenience, we divide the nanoribbon into three regions, i.e., the source (lead-*L*), the device, and the drain (lead-*R*). As a result, the transmission probability can be expressed as follows:

(3) $$T(\omega )=\text{Tr}[\Gamma_{L}(\omega )G_{D}^{r}(\omega )\Gamma_{R}(\omega )G_{D}^{a}(\omega )].$$

$$\Gamma_{L/R}=i(\Sigma_{L/R}-\Sigma_{L/R}^{†})$$

denotes the coupling between lead- *L* (*R*) and the device region, and Σ_*L*/*R*_ is the self-energy caused by the coupling between the device and lead regions. $G_{D}^{r/a}(\omega )$ represents the retarded/advanced Green function for the device region, which follows the relationship of *G*^*a*^ = [*G*^*r*^]^*†*^[42], $\Gamma_{L/R}=t_{T}^{2}g_{L/R}$ with *g*_*L*/*R*_ being the surface-state Green function of lead- *L*/*R*.

In general, *g*_*L*/*R*_ can be numerically solved with the iteration method. In this work, we would like to analytically solve them by projecting the semi-infinite AGNR in the Green function space into a semi-infinite one-dimensional double-atom chain [43]. By derivation, we get the coefficients of the Green function, i.e., $\left[W_{o}]=(\omega -\varepsilon_{c})I(N)-t_{0}^{2}(\omega -\varepsilon_{c}){\left[{\left(\omega -\varepsilon_{c}\right)}^{2}-t_{0}^{2}\right]}^{-1}[\Xi \right]$, $\left[W_{i}]=t_{0}^{3}(\omega -\varepsilon_{c}){\left[{\left(\omega -\varepsilon_{c}\right)}^{2}-t_{0}^{2}\right]}^{-1}[\Xi \right]$, and [*W*_*e*_] = *t*_0_*I* (*N*) are the onsite energy, the coupling between the two atoms in each primitive cell, and the coupling between the neighboring two primitive cells of the chain, respectively. If the AGNR width *M* is odd, $N=\frac{M-1}{2}$ and [*Ξ*]_*jl*_=2*δ*_*jl*_ + *δ*_*j*,*l* + 1_ + *δ*_*j*,*l* − 1_. Otherwise, $N=\frac{M}{2}$ and [*Ξ*]_*jl*_ = 2*δ*_*jl*_− *δ*_11_ + *δ*_*j*,*l* + 1_ + *δ*_*j*,*l*−1_. By diagonalizing matrix [*Ξ*], the double-atom chain can be transformed into its molecular orbit representation, and the surface state Green function can be expressed. After this, we can obtain the surface state Green function of the semi-infinite AGNR by representation transformation.

## Results and discussion

In this section, we aim to investigate the transport properties of this structure. Prior to calculation, we consider *t*_0_ to be the energy unit.

**Figure 1:**
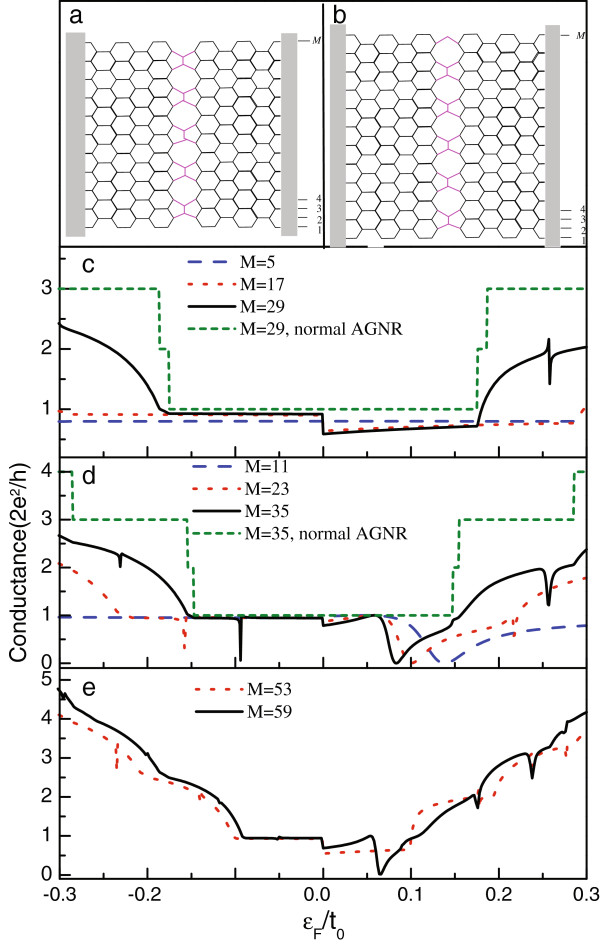
**AGNR widths.(a** and **b)** Schematics of AGNRs with line defect whose widths are *M* = 12 *n* − 7 and *M* = 12*n* − 1, respectively. **(c** to **e)** The linear conductance spectra of the different-width AGNRs with *M* = 5, 11, 17, 23, 29, 35, 53, and 59.

**Figure 2:**
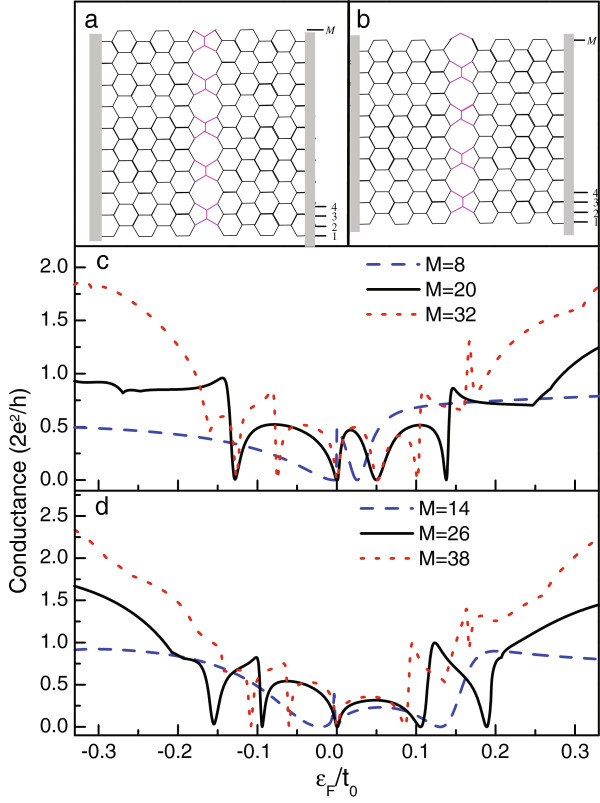
**AGNR configurations.(a** and **b)** Schematics of line defect-embedded AGNRs where *M* = 12*n*−4 and *M* = 12*n* + 2. **(c** and **d)** The linear conductance spectra of the AGNRs with *M* = 8, 14, 20, 26, 32, and 38.

When the graphene with line defect is tailored into an AGNR, one would find its various configurations. If one edge of the AGNR is perpendicular to the growth direction of the line defect and its profile is assumed to be unchanged, we will possess four different configurations, as shown in Figure 1a,b and Figure 2a,b. In Figure 1a,b, the AGNR widths are *M* = 12*n*−7 and *M* = 12*n* − 1, respectively. For the other configurations in Figure 2a,b, there will be *M*=12*n*−4 and *M* = 12*n* + 2. For convenience, we name the configurations illustrated in Figure 1a,b as model A and model B and those in Figure 2a,b as model C and model D, respectively. We first plot the linear conductance spectra of model A and model B in Figure 1c,d. The structure parameters are taken to be *ε*_*c*_ = *ε*_*d*_ = 0 and *t*_*T*_ = *t*_*D*_ = *t*_0_. It is obvious that independent of the configurations, the line defect suppresses the electron transport apparently. This is certainly attributed to the defect-contributed electron scattering. Moveover, one can find that the influence of the line defect is tightly determined by the AGNR configurations. In model A where *M*=12*n*−7, the first conductance plateau is suppressed, and the conductance magnitude deduces more obviously where *ε*_*F*_>0. However, the conductance plateau is still observed. With respect to the other conductance plateaus, they are destroyed seriously by the presence of line defect. For instance, when the AGNR width increases to *M*=29, conductance dips emerge in the vicinity of *ε*_*F*_ = 0.25*t*_0_ and *ε*_*F*_ = −0.3*t*_0_, respectively. For model B in which *M* = 12*n* − 1, in Figure 1d, one readily observes that the line defect modifies the electron transport in a different way. Namely, there always exists Fano antiresonance in the positive-energy region of the first conductance plateau, irrelevant to the width of the AGNR. With the increase of the AGNR width, the first conductance plateau becomes narrow; the antiresonance point accordingly shifts toward the Dirac point. Meanwhile, a conductance dip appears in the negative-energy region of the first conductance plateau. In order to compare the difference between these two models, we present the results of wide nanoribbons *M*=53 and *M* = 59 in Figure 1e. We do not find any new phenomenon except some conductance dips in the higher conductance plateaus.

In Figure 2c,d, we present the linear conductance spectra of model C and model D. The structure parameters are considered to be the same as those in Figure 1. It can be found that here, the Fano antiresonance becomes more distinct, including that at the Dirac point. Moreover, due to the Fano effect, the first conductance plateau almost vanishes. In Figure 2c where *M* = 12*n* − 4, we find that in the case of *M* = 8, one clear Fano antiresonance emerges at the Dirac point, and the wide antiresonance valley causes the decrease of the conductance magnitude in the negative-energy region. In addition, the other antiresonance occurs in the vicinity of *ε*_*F*_ = 0.03*t*_0_. When the AGNR widens to *M* = 20, the Fano antiresonances appear on both sides of the Dirac point respectively. It is seen, furthermore, that the Fano antiresonances in the positive-energy region are apparent, since there are two antiresonance points at the points of *ε*_*F*_ = 0.05*t*_0_ and *ε*_*F*_ = 0.14*t*_0_. Next, compared with the result of *M* = 20, new antiresonance appears around the position of *ε*_*F*_ = − 0.08*t*_0_ in the case of *M* = 32. In model D, where *M* = 12*n* + 2, the antiresonance is more apparent, in comparison with that of model C. For instance, when *M* = 14, a new antiresonance occurs in the vicinity of *ε*_*F*_ = 0.13*t*_0_, except the two antiresonances in the vicinity of the Dirac point. With the increase of *M* to *M* = 26, two antiresonance points emerge on either side of the Dirac point. However, in the case of *M* = 38, we find the different result; namely, there is only one antiresonance in the positive-energy region. This is because the widening of the AGNR will narrow the first conductance plateau. Consequently, when *ε*_*F*_ = 0.15*t*_0_, the Fermi level enters the second conductance plateau. In such a case, the dominant nonresonant tunneling of electron inevitably covers the Fano antiresonance.

**Figure 3:**
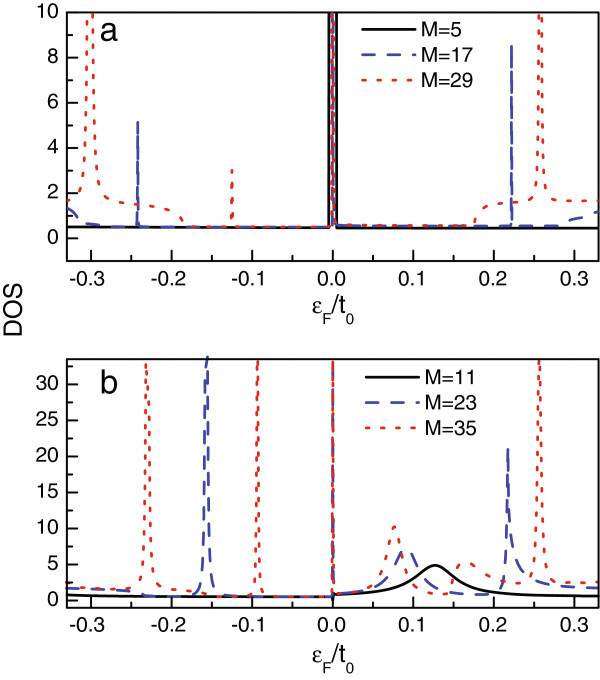
**The DOS of the AGNR with line defect.** In **(a)**, the widths of the AGNR are taken to be *M* = 5, 17, and 29. In **(b)**, *M* is equal to 11, 23, and 35, respectively.

The Fano antiresonance originates from the interference between one resonant and one nonresonant processes. It is thus understood that the line defect makes a contribution to the resonant electron transmission. Namely, the presence of line defect causes the appearance of new localized quantum state. The coupling between this localized state and the main transmission channel contributes to the resonant transmission. Surely, we should focus on the properties of the localized state to clarify the occurrence of the Fano antiresonance. Following this idea, we investigate the density of states (DOS) of such a structure. The numerical results of model A and B are shown in Figure 3a,b. By comparing the results in Figure 1 and Figure 3, we find that in the region where appears a conductance dip, the corresponding DOS spectrum shows up as a peak. This result exactly proves that the line defect induces the appearance of localized state which offers a resonant channel for the quantum interference. When the defect-induced state is less localized, the amplitude of the corresponding resonant path gets close to the nonresonant one; hence, the quantum interference is distinct, leading to the Fano antiresonance. Just as shown in Figure 3b, the widening of the quantum state is apparent around the point of *ε*_*F*_ = 0.1*t*_0_, so the Fano antiresonance is clearly observed in Figure 1d. In contrast, if the states are more localized, the quantum interference assisted by them is somewhat weak. Thus, one can only see the some weak conductance dips in the conductance curves. In addition, in Figure 3a,b, we can see that some DOS peaks do not correspond to the conductance dips in Figure 1c,d. One can ascertain that these states are completely localized and are decoupled from the main transmission channel. This is exactly called the BIC phenomenon [44].

**Figure 4:**
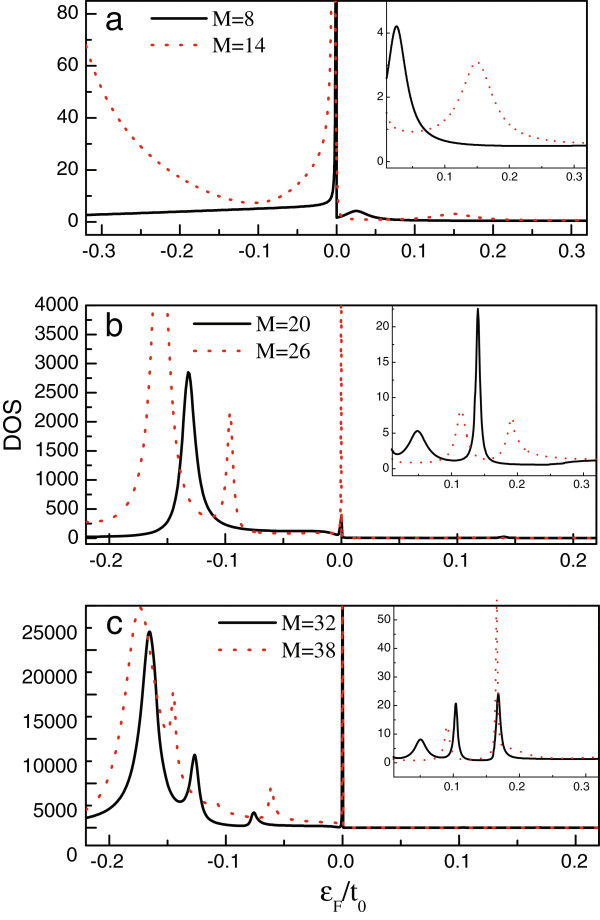
**The DOS of the AGNR with line defect.(a)** The widths of AGNR are taken to be *M* = 8 and 14. **(b)** The widths of AGNR are *M* = 20 and 26. In **(c)**, the values of *M* are 32 and 38, respectively.

The DOS spectra of model C and model D are shown in Figure 4a,c. Similar to the former two models, the DOS peaks are consistent with the Fano antiresonances in the conductance curves. Next, we find that the DOS peaks only distribute in the region of |*ε*_*F*_| < 0.2*t*_0_ with no peak in the other region. So, it is clearly known that the defect-induced localized states are confined in such a region in such two models. On the other hand, in these two models, the DOS peak around the Dirac point is wider (see Figure 4a). This leads to the apparent Fano antiresonance around the Dirac point. In addition, with the widening of the AGNR, the DOS spectra of the two models show similar variation behaviors. To be concrete, independent of the change of *M*, the DOS spectra on the two sides of the Dirac point exhibit completely different properties, and in the region of *ε*_*F*_ > 0, the amplitudes of the DOS peaks are much smaller than those in the region of *ε*_*F*_ < 0. It is also found that with the increase of *M*, the DOS peaks in the region of *ε*_*F*_ > 0 increase with the enhanced amplitudes of them. However, in the negative-energy region, when only *M* = 20, a strong DOS peak appears in the vicinity of *ε*_*F*_ = − 0.13 *t*_0_. Next, when the AGNR is further widened, such a peak enhances and splits obviously. The increase of the DOS peaks brings about the abundant Fano effects. Due to the enhanced DOS peaks in the negative-energy region, we can understand that the influence of the line defect is more evident in this region.

Following the above description, we next discuss the reason of the asymmetric DOS spectra of model C and model D. Note first that in the region of |*ε*_*F*_| → 0, [*W*_*o*_] ≈ *ε*_*F*_*I* (*N*) + *ε*_*F*_ [*Ξ*] and [*W*_*i*_] = − *tε*_*F*_ [*Ξ*]. It is evident that when *ε*_*F*_ > 0, the sign (+/−) of [*W*_*i*_]_*jl*_ is opposite to that of [*W*_*e*_]_*jl*_, whereas the signs of them are the same in the case of *ε*_*F*_ < 0. Such a result of electron-hole asymmetry certainly influences the surface state of the semi-infinite AGNR. Namely, when *ε*_*F*_ > 0, the surface state of the semi-infinite AGNR will become more localized. However, the line-defect Hamiltonian is of electron-hole symmetry. Hence, in the region of *ω* > 0, the electron transport is weaker than that in the region of *ω* < 0. Due to these reasons, we see that in the four models, the effect of the line defect in the negative energy is relatively weak. Next, in the even *M* case, [*W*_*o*_]_11_ ≈ 2*ε*_*F*_ and [*W*_*i*_]_11_ = −*tε*_*F*_ in the region of |*ε*_*F*_| → 0. This will modify the surface state properties of the semi-infinite nanoribbon. With the help of the method offered in [43], we have found that in the case of even *M*, the surface state of the semi-infinite nanoribbon can be further localized in the case of *ε*_*F*_ > 0. Consequently, in such a case, the imaginary part of the self-energy contributed by the semi-infinite AGNR becomes small. Therefore, we can understand the reason for the asymmetric DOS states in model C and model D above and below the Dirac point.

**Figure 5:**
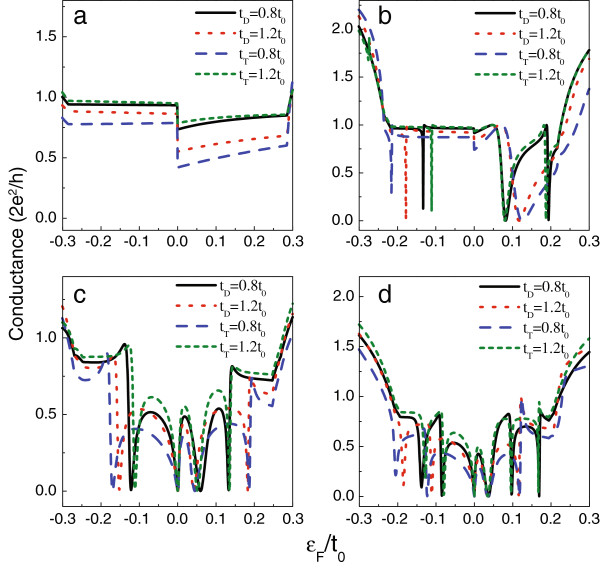
**The effect of the change of*t***_***d***_** and*t***_***T***_** on the AGNR conductance.** In **(a** to **d)**, *M* is taken to be 17, 23, 20, and 26, respectively.

Based on the previous works, the tight-binding results are consistent with those based on the density functional theory (DFT) calculations [40]; however, the values of *t*_*D*_ and *t*_*T*_ are certainly different from *t*_0_ due to the defect-induced change of the topological structure of the AGNR. Next, we would like to investigate the conductance affected by the deviation of the line-defect intersite coupling (*t*_*D*_) and the coupling between the defect and the AGNR (*t*_*T*_) from *t*_0_. We take model A with *M* = 17, model B with *M* = 23, model C with *M* = 20, and model D with *M* = 26 to calculate the change of linear conductance by the varied *t*_*D*_ and *t*_*T*_. The numerical results are shown in Figure 5. We see that the variation of *t*_*D*_ and *t*_*T*_ indeed adjusts the electron transport. In Figure 5a, when *t*_*D*_ increases on the two sides of the Dirac point, the difference between the conductance values is enlarged, leading to the further asymmetry of electron transport. With respect to the other models, the changes of the conductance spectra are mainly manifested as the shift of the Fano antiresonances. First, for model B and model C, Figure 5b,c shows that the decrease of *t*_*D*_ (or the increase of *t*_*T*_) causes the Fano antiresonances to shift to the Dirac point. In the opposite case, the Fano antiresonances on the two sides of the Dirac point will repel each other. For model D, the shift of Fano antiresonances exhibits different results. We see that the decrease of *t*_*D*_ (or the increase of *t*_*T*_) causes the Fano antiresonances to shift right, whereas the Fano antiresonances shift left under the opposite situation. Albeit the shift of conductance spectra, the conductance properties can not be basically modified.

**Figure 6:**
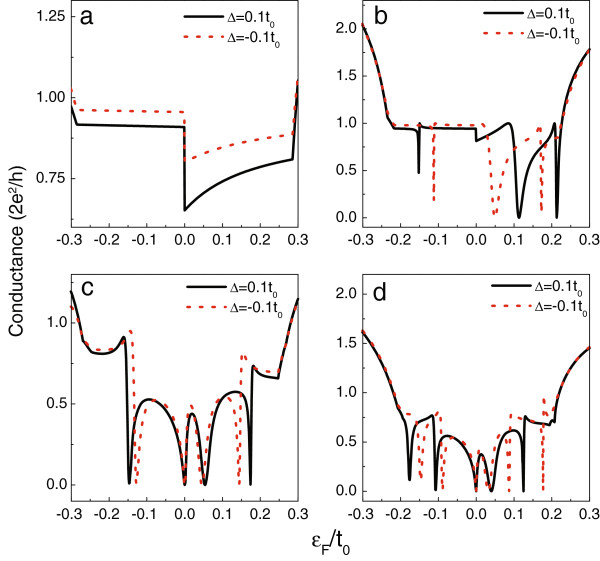
**The linear conductance of AGNR with the changed defect onsite energy.** In **(a** to **d)**, *M* is equal to 17, 23, 20, and 26, respectively.

When the line defect is embedded in the GNR, its onsite energy may be different from that of the GNR. Thus, in Figure 6, we present the influence of the change of the onsite energy of the line defect by taking *ε*_*d*_ = *ε*_*c*_ + *Δ*. For model A, in the case of positive *Δ*, the conductance magnitude decreases more apparently in the positive-energy region, as shown in Figure 6a. For the other models, the Fano antiresonances will depart from their original positions, except those at the Dirac point. In Figure 6b,c, when a positive *Δ* is considered, the Fano antiresonances in the region of *ε*_*F*_ > 0 shift to the high-energy direction, but those in the region of *ε*_*F*_ < 0 will move to the low-energy direction. Alternatively, when *Δ* is negative, the Fano antiresonance shifts to the Dirac point. As for the results about model D, Figure 6 shows that the positive *Δ* causes the Fano antiresonances to shift left, whereas the Fano antiresonances shift right in the presence of a negative *Δ*. Up to now, we find that the deviations of the onsite energy, *t*_*D*_, and *t*_*T*_ induce the similar change of the conductance spectra. It should be pointed out that in spite of the shift of the conductance spectra, the main conductance properties assisted by the line defect are robust. According to these calculations, the contribution of the line defect to the electron transport in the AGNR can be well understood.

## Conclusion

In summary, we have investigated the electron transport through an AGNR with line defect from the theoretical aspect. As a consequence, it has been found that the line defect induces the Fano effects or the phenomenon of BIC in electron transport through this structure, which are determined by the width of the AGNR. To be specific, when *M*=12*n*−7 or *M* = 12*n*−1, the Fano effects are comparatively weak, whereas the result of BIC is abundant. However, in the configurations of *M* = 12*n*−4 or *M* = 12*n*+2, the Fano effects are dominant, and no BIC phenomenon has been observed. By paying attention to the DOS spectra, we saw that the line defect induces some localized quantum states around the Dirac point and that the different localizations of these states lead to these two transport results. Next, the influences of the changed structure parameters on the Fano effects have been presented. We believe that the numerical results are helpful for clarifying the contribution of the line defect to the electron transport in the AGNR. We propose such a structure to be a promising candidate for nanoswitch.

## Competing interests

The authors declare that they have no competing interests.

## Authors’ contributions

WJG designed the theoretical model, deduced the relevant formula, and drafted the manuscript. XYS and YW carried out the numerical calculations. GDY participated in the analysis about the results. XHC improved the manuscript. All authors read and approved the final manuscript.
